# Magnetically-actuated hydrogel-based achiral planar microswimmers for SERS detection: In situ coprecipitation for continuous loading of iron oxide nanoparticles

**DOI:** 10.3389/fbioe.2023.1086106

**Published:** 2023-03-07

**Authors:** Junfeng Xiong, Junkai Zhang, Yukun Zhong, Xiaoxia Song, Haoying Wang, U Kei Cheang

**Affiliations:** ^1^ School of Mechatronics Engineering, Harbin Institute of Technology, Harbin, China; ^2^ Department of Mechanical and Energy Engineering, Southern University of Science and Technology, Shenzhen, China; ^3^ Shenzhen Key Laboratory of Biomimetic Robotics and Intelligent Systems, Southern University of Science and Technology, Shenzhen, China; ^4^ Guangdong Provincial Key Laboratory of Human-Augmentation and Rehabilitation Robotics in Universities, Southern University of Science and Technology, Shenzhen, China

**Keywords:** SERS detection, *in situ* coprecipitation, ultraviolet lithography, achiral planar microswimmers, magnetic control

## Abstract

Ultraviolet lithography is a very promising technology used for the batch fabrication of biomedical microswimmers. However, creating microswimmers that can swim at low Reynolds number using biocompatible materials while retaining strong magnetic properties and excellent biomedical functionality is a great challenge. Most of the previously reported biomedical microswimmers possess either strong magnetic properties by using non-biocompatible nickel coating or good biocompatibility by using iron oxide particle-embedded hydrogel with weak magnetism, but not both. Alternatively, iron oxide nanoparticles can be coated on the surface of microswimmers to improve magnetic properties; however, this method limited the usability of the microswimmers’ surfaces. To address these shortcomings, this work utilized an *in situ* synthesis technique to generate high magnetic content inside hydrogel-based achiral planar microswimmers while leaving their surfaces free to be functionalized for SERS detection. The hydrogel matrices of the magnetically actuated hydrogel-based microswimmers were first prepared by ultraviolet lithography. Then, the high concentration of iron oxide was achieved through multiple continuous *in situ* coprecipitation cycles. Finally, the SERS detection capability of magnetically actuated hydrogel-based microswimmers was enabled by uniformly growing silver nanoparticles on the surface of the microswimmers. In the motion control tests, the microswimmers showed a high swimming efficiency, high step-out frequency, and consistent synchronized motion. Furthermore, the magnetically actuated hydrogel-based microswimmers were able to improve the detection efficiency of analytes under magnetic guidance.

## 1 Introduction

With the development of micro- and nanofabrication technologies, many viable techniques can be used to fabricate microswimmers, such as biotemplating synthesis (Zhang et al., 2009; Schuerle et al., 2012; Gao et al., 2014; Ali et al., 2017), chemical synthesis (Cheang et al., 2017), lithography (Chen et al., 2021; Mu et al., 2021; Xiong et al., 2022), and 3D printing (Wang et al., 2018; Dong et al., 2020; Lee et al., 2021; Park et al., 2021). Among these techniques, ultraviolet lithography (UVL) and thin-film deposition technologies demonstrated the advantages of using parallel batch fabrication to achieve high efficiency, high consistency, and low-cost manufacturing of microswimmers (Zhang et al., 2009; Huang et al., 2016; Go et al., 2018; Tottori and Nelson, 2018; Xu et al., 2019; Mu et al., 2021). Most microswimmers created using these techniques were coated with a nanolayer of nickel for magnetic actuation; however, nickel is not biocompatible and may present problems if used for *in vivo* applications. For example, nickel may damage cell wall integrity and can cause adverse health effects, including cardiovascular, kidney, and lung diseases (Genchi et al., 2020). Therefore, direct exposure of nickel should be avoided as much as possible. In addition, the compact nickel nanolayer may hinder surface functionalization, which further reduces its application. Thus, there is a need to find alternative magnetic materials that can maintain both the biocompatibility and functions of microswimmers. Iron oxide is an ideal magnetic material for *in vivo* applications because of its low toxicity (Mahmoudi et al., 2012). For example, by embedding superparamagnetic iron oxide nanoparticles into the hydrogel matrix, Hakan *et al.* were able to fabricate biocompatible magnetic helical microswimmers using two-photon polymerization (TPP) for theranostic cargo delivery (Ceylan et al., 2019). However, the content of iron oxide nanoparticles in the microswimmers was limited because a high concentration of iron oxide nanoparticles prevents the photocuring of hydrogel which can affect the structural stability of the microswimmers; thus, the helical microswimmers had relatively weak magnetic moments, leading to a low step-out frequency of 5 Hz and swimming efficiency of 0.028 when actuated by a 20-mT rotating magnetic field (RMF). Later, Lee et al. (2021) fabricated magnetically actuated drug delivery helical microswimmers using a similar way and obtained a step-out frequency of 4 Hz under a 15-mT RMF; the reason for the relatively low step-out frequency was the low concentration of iron oxide nanoparticles. Dong et al. (2020) incorporated magnetic nanoparticles into the microswimmers by immersing the microswimmers in an iron-based nanoparticle solution; as a result, the microswimmers were coated with a layer of nanoparticles and were able to reach a higher step-out frequency of 32 Hz and a swimming efficiency of 0.053 under a 3-mT RMF. However, this approach completely covered the surface of the hydrogel microswimmers, which hindered the possibility of further functionalization of the microswimmers’ surfaces.

To overcome the aforementioned shortcomings, this work introduces the magnetically actuated hydrogel-based achiral planar microswimmers (MHMs) with high iron oxide content for SERS detection. In this work, the hydrogel matrices (HMs) of the MHMs were prepared by UVL. Then, iron oxide nanoparticles were grown inside the HMs through *in situ* coprecipitation to create MHMs. Compared to previous studies, where iron oxide nanoparticles were embedded inside the bodies of the microswimmers, repeated *in situ* coprecipitation yielded MHMs with significantly enhanced magnetic properties by increasing the iron oxide content. As a result of the high magnetic content and their achiral planar structures, the MHMs showed a high step-out frequency and swimming efficiency. The MHMs also showed consistent synchronized motion during the motion control tests. To increase the usability of the MHMs, silver nanoparticles were uniformly grown on the surface of the MHMs through the silver mirror reaction to create MHM probes with SERS detection capability. The silver nanoparticles enhanced the SERS signal and facilitated the detection of the analyte with high sensitivity. This is demonstrative of how the fabrication method used in this work allowed the surface of the hydrogel to be free for functionalization. Furthermore, the magnetically actuated propulsive motion of the MHMs can facilitate contact between the silver nanoparticles and the analyte, which improved the detection efficiency of the MHM probes. The detection efficiency of the analyte of the MHM probes in the active state was positively correlated with the rotation speed of the MHM probes. For chemically driven micromotor-based Raman detection, high fuel concentration led to the oxidation deactivation of analyte molecules, which instead resulted in the reduction of Raman strength. Compared to chemically driven microswimmer-based Raman detection (Orozco et al., 2013; Han et al., 2016), the MHM probes have the advantage of being non-destructive to the analyte molecules. In short, this work demonstrated that the synergy between UVL of hydrogel, *in situ* coprecipitation, surface functionalization, and magnetic actuation allowed for an efficient way to mass manufacture highly functional microswimmers.

## 2 Materials and methods

### 2.1 Materials

Acrylic acid (AA), trimethylolpropane ethoxylate triacrylate (Mn = 428 g mol^−1^, PEG_428_-triacrylate), polyvinylpyrrolidone (Mn = 360,000 g mol^−1^, PVP), 2,2-dimethoxy-2-phenylacetophenone (DMPA), dimethylformamide (DMF), isopropyl alcohol (IPA), sodium hydroxide (NaOH), ferric chloride hexahydrate (FeCl_3_·6H_2_O), ferrous chloride tetrahydrate (FeCl_2_·4H_2_O), ammonium hydroxide (NH_3_·H_2_O), and silver nitrate solution were purchased from Aladdin.

The material composition of the initial pre-polymer mixture used to create the HMs consisted of 15 wt% AA, 15 wt% PEG_428_-triacrylate, 10 wt% PVP, 3 wt% DMPA, and 57 wt% DMF.

### 2.2 Fabrication of the HMs

**FIGURE 1:**
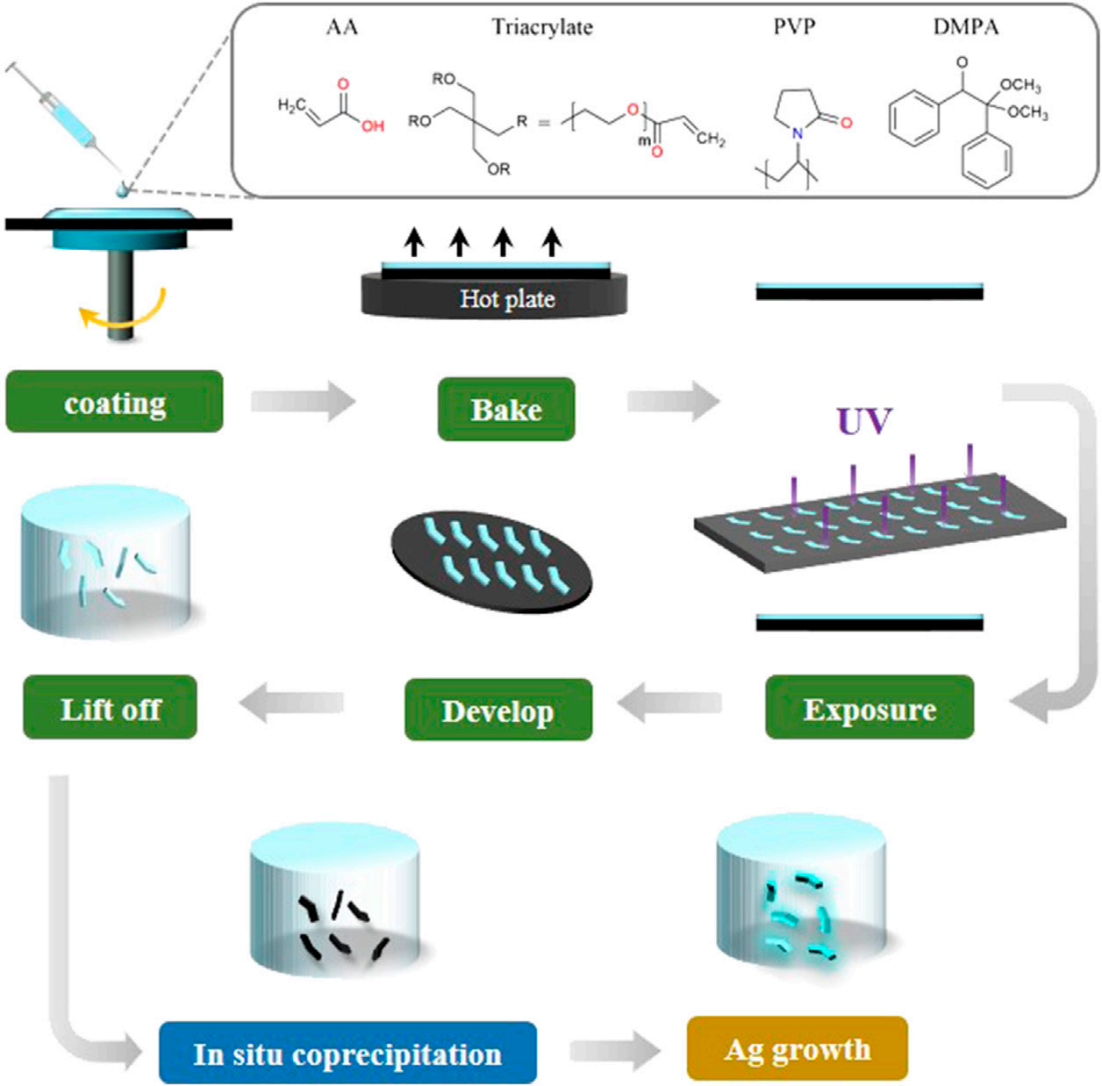
Schematics of the fabrication procedure of the MHMs.

The HMs were prepared by UV-initiated free-radical polymerization of acrylate in the pre-polymer mixture. The HMs were fabricated using UVL, as shown in Figure 1. First, a uniform layer of the pre-polymer mixture was formed by spinning 3 mL of the mixture onto a silicon wafer at 1,000 rpm for 60 s. The wafer was then prebaked at 95°C for 3 min to evaporate the organic solvent DMF completely, which resulted in a homogeneous, hard film on the wafer. The condensed long PVP chain acts as a scaffold to prevent PEG_428_-triacrylate from flowing freely. After cooling to room temperature, the hard film was exposed to UV light (Mask Aligner, SUSS MA6, Germany) for 0.2 s at 8.9 mW cm^−2^ through a chrome photomask. The photoinitiator DMPA initiates PEG_428_-triacrylate cross-linking polymerization under UV light, and PVP and PEG polymer networks were formed with permanent polymerization structures. After exposure, the HMs were developed by immersing the wafer in IPA to wash away the unpolymerized region to reveal the polymerized HMs. The HMs were washed three times with DI water and visually examined under an inverted optical microscope (MJ33, Mingmei, China).

### 2.3 Fabrication of the MHMs: *In situ* magnetic nanoparticle synthesis

*In situ* coprecipitation was previously used to grow iron oxide nanoparticles inside the microparticles (Suh et al., 2012). Here, a similar method was used to grow iron oxide nanoparticles inside the HMs. To fully deprotonate the COOH groups of AA to COO^−^ groups, the HMs were dispersed in 0.5 M NaOH solution for 15 min and washed three times with DI water to remove excess NaOH solution. Then, 0.2 M FeCl_3_ and 1 M FeCl_2_ solutions were mixed in a ratio of 1:15 with the HMs for 20 min. After the iron ions diffused into the HMs and fully formed a chelate with COO^−^ groups, the excess solution was removed by using an absorbent paper. NH_3_·H_2_O and DI water were mixed in a ratio of 1:2 with the HMs at 65°C for 15 mins, resulting in iron oxide nanoparticles nucleated and growing inside the HMs, which yielded the MHMs. The MHMs were washed three times with DI water. To further increase the magnetic content, the *in situ* coprecipitation process was repeated five times.

### 2.4 Fabrication of the MHM probes for SERS

A layer of silver nanoparticles was grown on the surface of the MHMs through the silver mirror reaction to enable SERS detection capability (Wang, 2020). First, 1 g of PVP was dissolved in 10 mL of ethanol. Then, 200 μL of silver nitrate solution and 100 uL of the MHM solution were added to the mixture and shaken for 5 min. Finally, the mixture was transferred to a reaction kettle and heated at 160°C for 4 h. After transfer to room temperature, the MHM probes were collected by centrifugation (5,000 r/min for 3 min) and washed three times with DI water.

### 2.5 Characterization

The size and surface morphology of the HMs, MHMs, and MHM probes were characterized using scanning electron microscopy (SEM, Merlin, ZEISS, Germany) at 2 keV. The elemental composition of the MHM probes was characterized using energy-dispersive spectrometry (EDS, Octane Pro, United States) at 5 keV. The magnetic hysteresis loop of the MHMs was obtained using a vibrating sample magnetometer (VSM, 7404, Lake Shore, United States) at room temperature.

### 2.6 Motion control test

The MHMs were actuated using a control system composed of an imaging system (microscope and camera) and a three-dimensional Helmholtz coil system. For motion control test, 300 μL of the solution containing the MHMs was dropped onto a sealed PDMS chamber. A 10-mT RMF produced by a magnetic coil system was used to manipulate the MHMs. By adjusting the rotation frequency and direction of the RMF, the propulsive velocity of the MHMs can be adjusted. The imaging system recorded their movement at 30 fps, and a MATLAB tracking algorithm was used to analyze the propulsive velocity and trajectory of the MHMs.

### 2.7 SERS detection of crystal violet

As shown in Figure 5, 50 μL of crystal violet (CV) ethanol solution with a gradient concentration of 10^–5^ ∼ 10^–8^ M was first dropped into a PDMS chamber. Then, 50 μL of ethanol containing the MHM probes was then transferred to the CV solution for molecular adsorption. After 60 min of molecular enrichment, excess CV solution was removed, and the MHM probes were dried for Raman measurements. The Raman spectra were collected using a confocal Raman spectrometer (LabRAM HR Evolution, Horiba, Japan) with a 532 nm laser and an integration time of 30 s. As shown in Figure 6B, the active MHM probes were actuated by a 15-Hz RMF for active molecular adsorption. As shown in Figure 6D, the active MHM probes were actuated by a 2-Hz, 5-Hz, and 10-Hz RMF.

## 3 Results

### 3.1 Fabrication and characterization of MHMs

**FIGURE 2:**
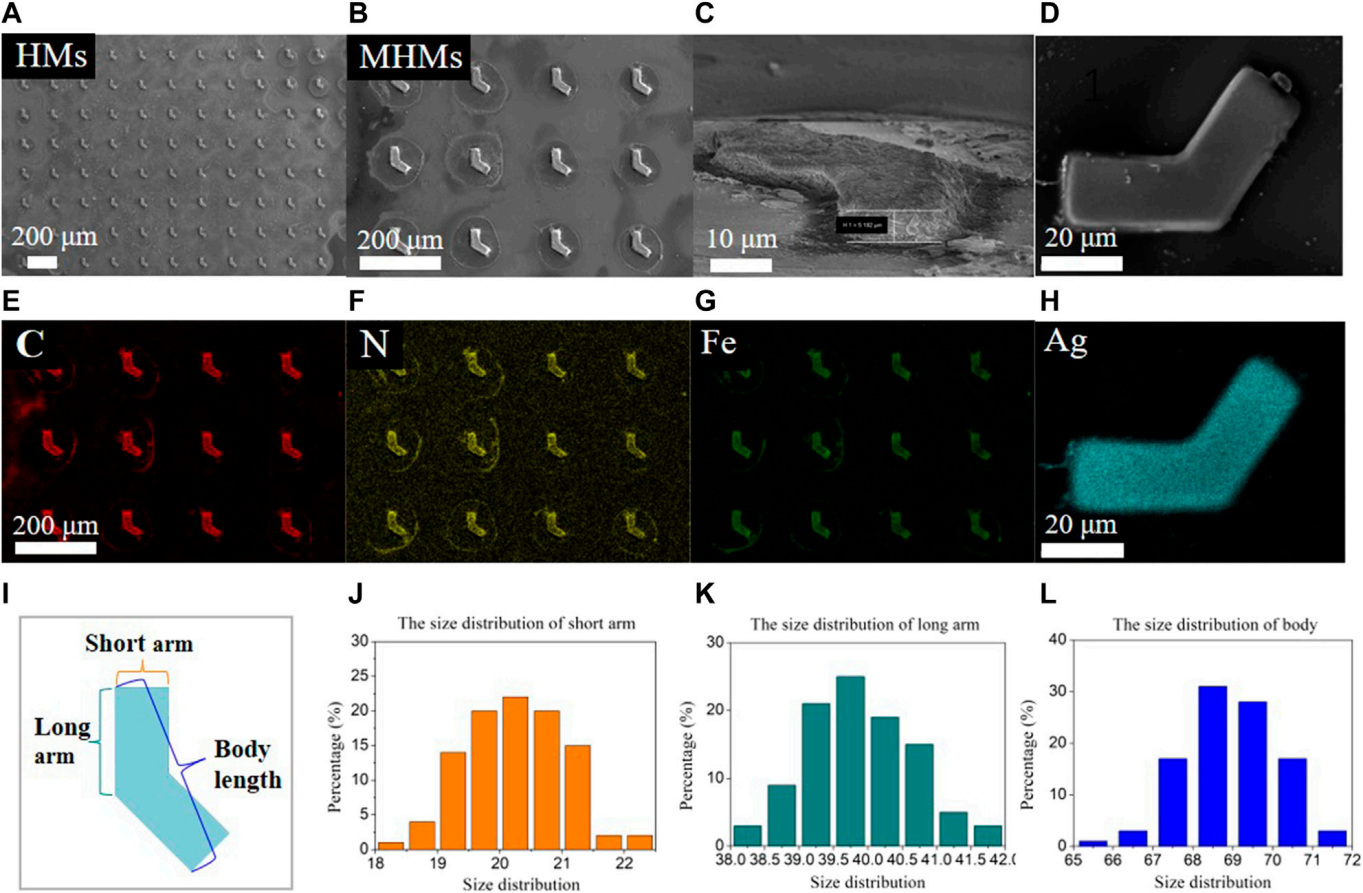
Physical characterization and size distribution of the microstructures. SEM images of **(A)** HMs, **(B,C)** MHMs, and **(D)** an MHM probe with Ag nanoparticles, respectively. EDS elemental mapping of **(E–G)** MHMs and **(H)** an MHM probe. **(I–L)** Size distribution of the short arm, long arm, and body length.

The MHM probes were carefully examined at the three major stages of fabrication: HMs after UVL, MHMs after *in situ* coprecipitation, and MHM probes after the silver mirror reaction. Their morphology and elemental composition were characterized using SEM/EDS. The SEM images of HMs, MHMs, and MHM probes are shown in Figure 2A–D, respectively. EDS mapping showed the uniform distribution of C\N\Fe in the MHM after the *in situ* coprecipitation process (Figures 2E–G) and the uniform distribution of silver nanoparticles on the MHM probes (Figure 2H). The uniform coating of silver nanoparticles enables the function of SERS detection. To verify the consistency of the UVL fabrication of the HMs, the size distributions of the HMs were measured, which included the distribution of short arms, long arms, and body lengths (Figure 2I). As shown in Figures 2J–L, the size distributions of the short arm, long arm, and body lengths were 20.2 ± 0.7 μm, 39.9 ± 0.8 μm, and 69.0 ± 1.2 μm, respectively. On a single substrate, 280,000 HMs can be obtained from 3 mL of the pre-polymer mixture, which indicates the high manufacturability of this method. These experimental results demonstrated that the UVL process can create MHM probes with high uniformity, efficiency, and consistency.

### 3.2 *In situ* magnetic nanoparticle synthesis

**FIGURE 3:**
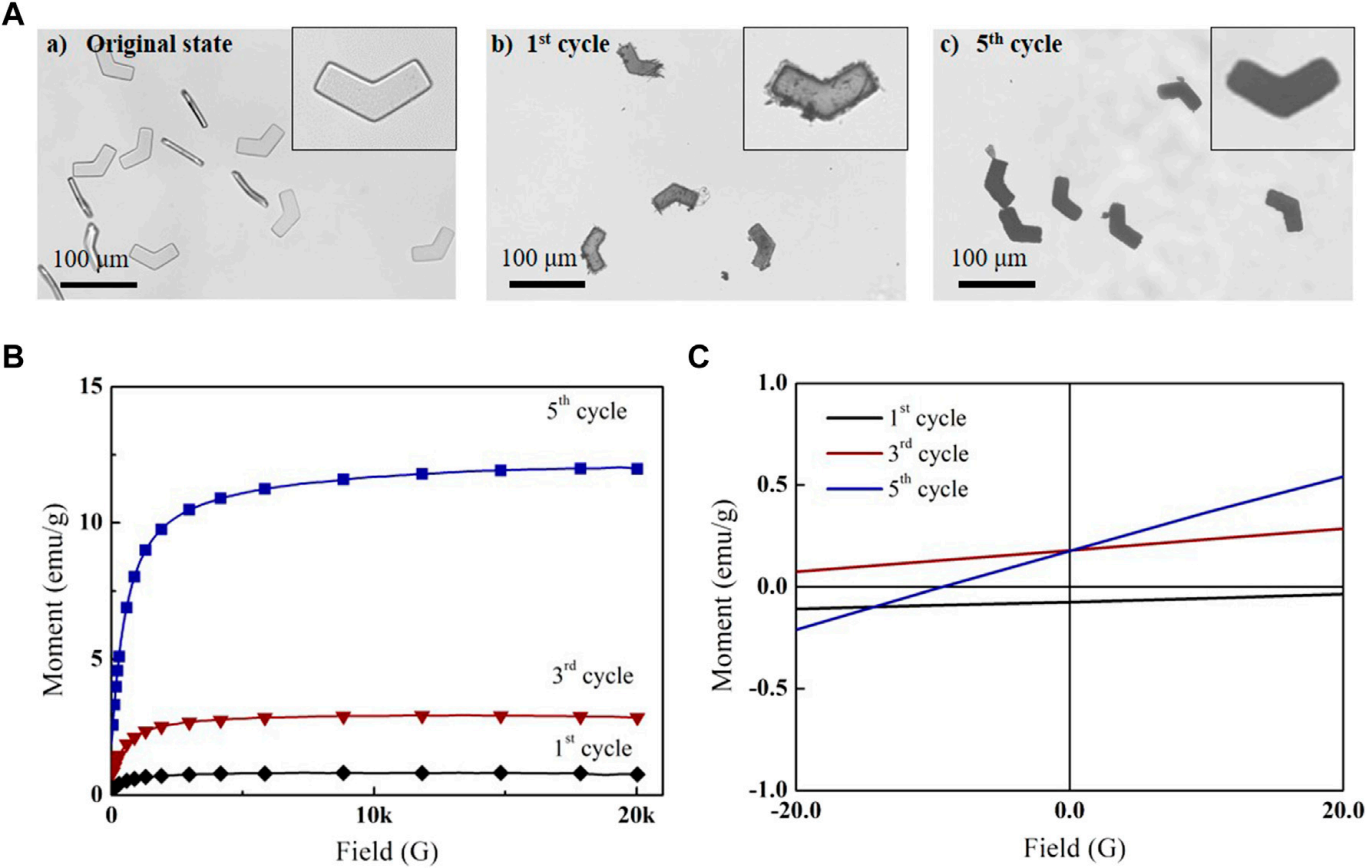
*In situ* coprecipitation of magnetic nanoparticles in MHMs. **(A)** Successive synthesis of magnetic nanoparticles in MHMs after **(a)** first, **(b)** third, and **(c)** fifth cycles. **(B)** Magnetization curves of the MHMs. **(C)** Magnetization values of the MHMs under the ± 20-G magnetic field.

The *in situ* coprecipitation method was used to grow iron oxide nanoparticles inside the MHMs. Several *in situ* coprecipitation cycles were performed to increase iron oxide nanoparticle content; the morphology and magnetic properties of MHMs were tested after several cycles. The optical images of HMs before *in situ* coprecipitation, MHMs after the first cycle, and MHMs after the fifth cycle are shown in Figures 3A(a–c). The results showed that the MHMs gradually darkened with the increase in the number of cycles, which suggests that more iron oxide nanoparticles were grown. For quantitative analysis of the magnetic property of the MHMs after *in situ* coprecipitation, the hysteresis loops of the MHMs were obtained using a VSM (Figure 3B). After the first, third, and fifth cycles, the saturation magnetizations of the MHMs reached 0.7, 3.3, and 12.0 emu/g, respectively, which indicates that multiple cycles can enhance the magnetism of the MHMs. In addition, Figure 3C shows that the magnetization values of the MHMs are close to zero when no magnetic field is applied, which indicates that the synthesized iron oxide nanoparticles are superparamagnetic. To verify the biocompatibility of the MHMs loaded with iron oxide nanoparticles, cell survival experiments were carried out, and the results showed that MHMs had very low toxicity to cells (Supplementary Figure S1).

### 3.3 Magnetic actuation of MHMs

**FIGURE 4:**
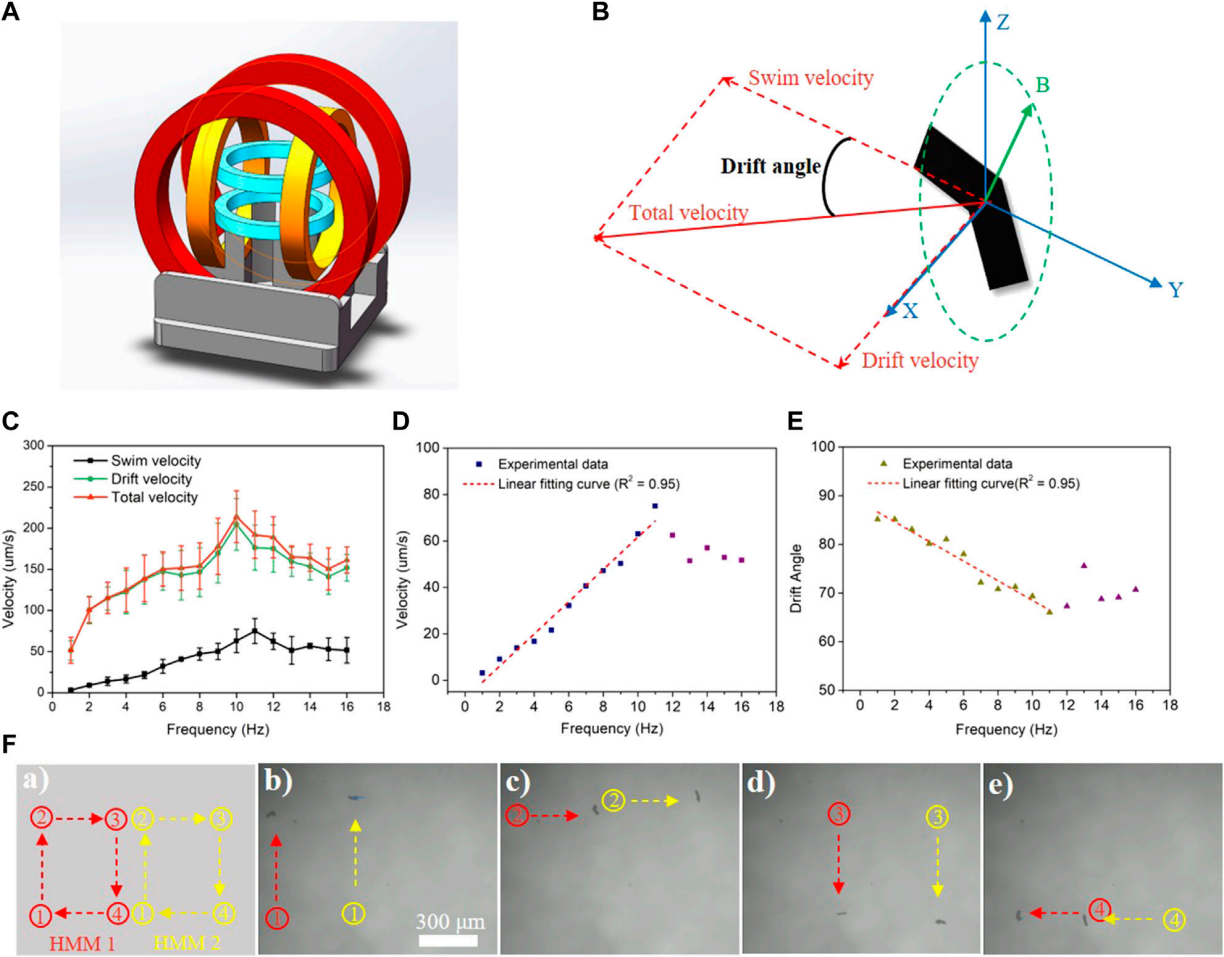
**(A)** Schematic illustration of the magnetic coil system. **(B)** Schematic of swimming and drifting directions of the MHM relative to the RMF shown in Cartesian coordinates. **(C–E)** Swimming, drift, and total velocity profiles of the MHMs vs frequency under a 10-mT RMF. The error represents the standard error with a sample size of four. **(F)** Synchronous movement of two MHMs under a 10-mT RMF at 6 Hz.

MHMs are capable of precise movement guided by an external RMF generated using a magnetic coil system (Figure 4A) (Cheang et al., 2017; Sachs et al., 2018). The motion schematic of a representative MHM is shown in Figure 4B. The RMF actuated the MHMs by generating a magnetic torque that drove the MHMs to rotate synchronously with the RMF; this allows the MHMs to convert rotational motion into translational motion. Since the MHMs were close to the bottom of the PDMS chamber, their total velocity consisted of two components: swimming velocity, *V*_*s*_, and drifting velocity, *V*_*d*_ (Figure 4B). The drifting motion was due to the interaction with the substrate (Peters et al., 2014).

The relationship between the velocity of MHMs and the RMF frequency is shown in Figure 4C (also see Supplementary Video S1). As the frequency of the RMF increased, the resultant velocity ( $V_{r}=\sqrt{V_{s}^{2}+V_{d}^{2}}$ ) and *V*_*d*_ increased significantly. The *V*_*r*_ of MHMs reached the maximum value of 214 μm/s (∼3.1 body length/s) at 10 Hz. After 10 Hz, *V*_*r*_ decreased slowly but the MHMs maintained a steady rotation. Interestingly, this is different from the direct loss of motion ability after step-out for rigid microswimmers. We speculated the reason to be subtle deformations of their soft bodies (unobservable from Supplementary Video S1), leading to this behavior after max velocity. The relationship between *V*_*s*_ of the MHMs and the RMF frequency is shown in Figure 4D. The average *V*_*s*_ profile is highly linear from 1 to 11 Hz, and the maximum *V*_*s*_ reached was 74.8 μm/s at 11 Hz. The maximum average swimming efficiency was 0.1 (swimming efficiency = *V*_*s*,__*max*_/(*Lf*), *V*_*s*,__*max*_ = 74.8 μm/s, body length *L* = 69.0 µm, and frequency *f* = 11 Hz). Supplementary Table S1 summarizes the parameters of the magnetically actuated hydrogel-based microswimmers reported in the last 5 years. Supplementary Table S1 shows that the MHMs have higher step-out frequency than many of the previously reported magnetically actuated hydrogel-based microswimmers that used physical mixing or chemical conjugation to incorporate magnetic material (Ceylan et al., 2019; Park et al., 2019; Lee et al., 2021; Park et al., 2021). Microswimmers that utilized surface coating of magnetic nanoparticles have higher step-out frequencies (Wang et al., 2018; Dong et al., 2020); however, their surfaces are completely covered which may hinder further functionalization. Furthermore, the MHMs have a swimming efficiency on par with the other types of microswimmers (Wang et al., 2018; Ceylan et al., 2019; Park et al., 2019; Dong et al., 2020; Lee et al., 2021; Park et al., 2021). The high step-out frequency of the MHMs was due to the increase in iron oxide content from the *in situ* coprecipitation process that allowed them to have a larger magnetic moment. This comparison highlighted that *in situ* synthesis offers a way to increase the magnetic moment of the hydrogel microswimmers without modifying the surfaces of the microswimmers. Furthermore, we also analyzed the drift angle [*θ* = cos^−1^(*V*_*s*_/*V*_*r*_)], which represents the influence of the surface on the swimming state of the MHMs. As shown in Figure 4E, the drift angle decreased linearly as the frequency increased. This indicates that the MHMs are less affected by the interaction with the substrate at higher frequencies. In addition, the synchronized motion of two MHMs under an RMF (10 mT, 6 Hz) is shown in Figure 4F (also see Supplementary Video S2). The red and yellow curves represent the trajectories of MHM1 and MHM2, respectively, as shown in Figure 4F(a). As shown in Figures 4F(b–e), the two MHMs can move along their respective square trajectories under the precise control of the RMF.

### 3.4 SERS sensing of the MHMs.

**FIGURE 5:**
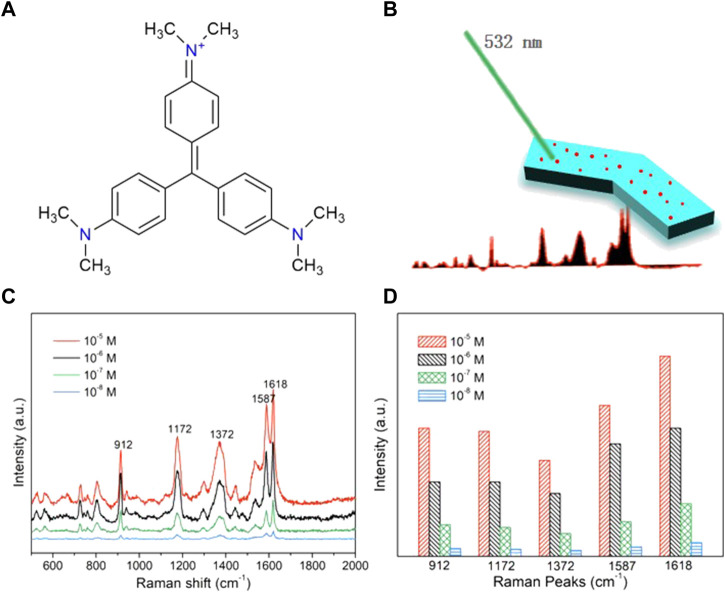
SERS sensing of the MHMs. **(A)** Chemical formula of the CV molecules. **(B)** Schematic illustration of SERS sensing of the MHMs. **(C)** SERS spectra of CV were acquired from MHMs with different concentrations of CV. The five characteristic Raman peaks of CV are marked at 912, 1,172, 1,372, 1,587, and 1,618 cm^−1^. **(D)** Raman intensity histograms of the five characteristic Raman peaks derived from the SERS spectra.

To demonstrate that the *in situ* coprecipitation can yield MHMs with surfaces that are free to be functionalized for different applications, silver nanoparticles were grown on the surfaces of the MHMs to enable SERS detection capability. Silver nanoparticles are a common, highly active, and biocompatible SERS substrate (Liu et al., 2009). They can enhance the Raman spectrum signal of analyte molecules and improve the detection sensitivity of the analyte, which is very suitable for biological analysis. To test the SERS sensing ability of the MHM probes, CV was selected as the analyte. CV is a commonly used analyte, and its chemical structure is shown in Figure 5A. The main Raman peaks of CV are observed at 912, 1,172, 1,372, 1,587, and 1,618 cm^−1^ (Raman peaks and corresponding vibrational functional groups are shown in Supplementary Table S2) (Liu et al., 2009). First, the MHM probes were immersed in CV ethanol solution at different concentrations (10^–5^ ∼ 10^–8^ M) for 60 min. Then, the Raman spectrum of CV was collected by laser Raman spectroscopy to analyze the relationship between concentration and Raman intensity, as shown in Figure 5B. The peak position of the Raman spectrum was almost consistent with the peak position corresponding to vibration functional groups in Supplementary Table S2, verifying the SERS capability of the MHM probes (Figure 5C). The variation in Raman intensity at different CV concentrations at the same peak position is shown in Figure 5D. As the CV concentration gradually decreased (10^–5^ ∼ 10^–8^ M), the intensity of all peaks gradually decreased, indicating that the detection ability of CV by MHM probes is positively correlated with CV concentration.

**FIGURE 6:**
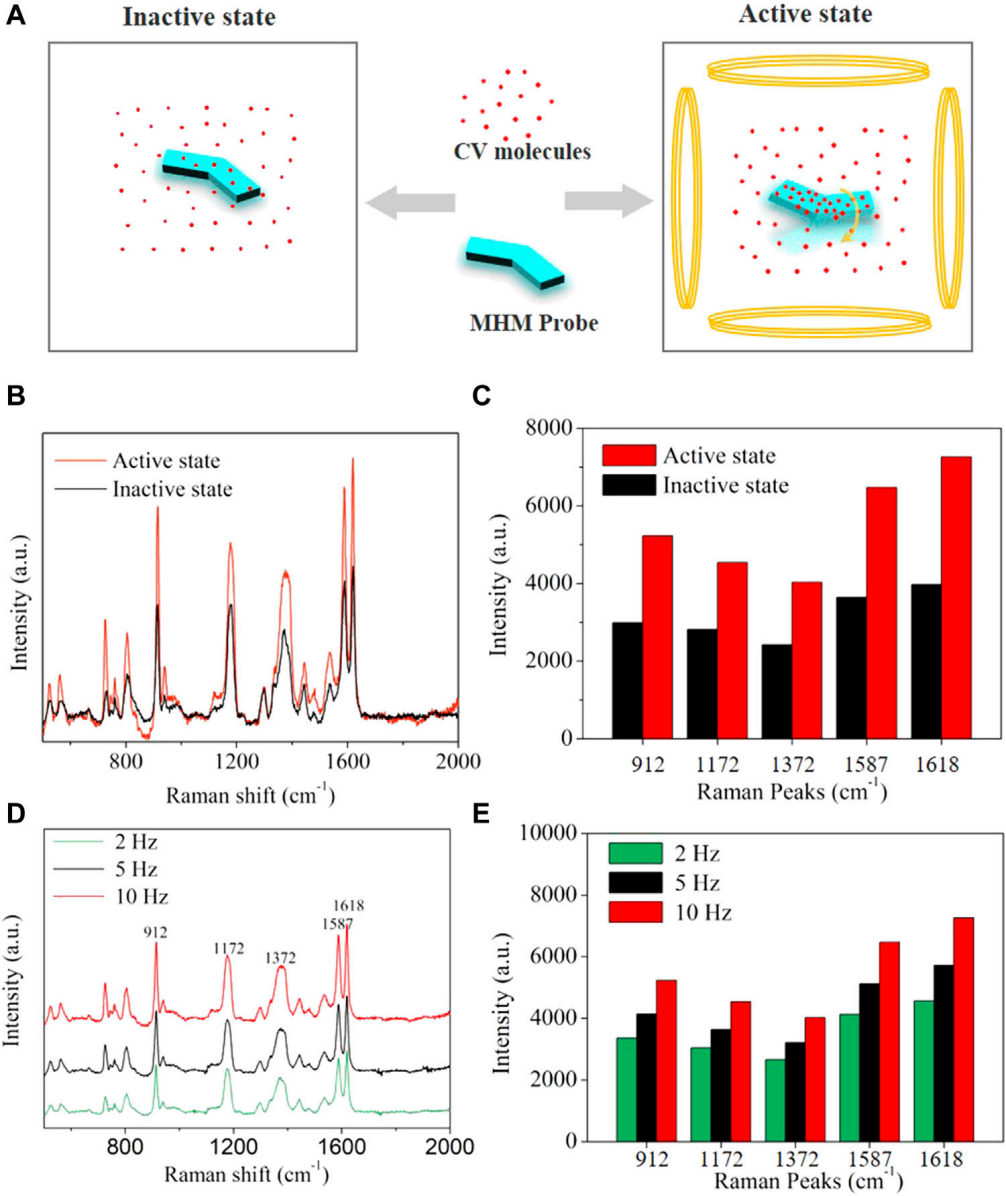
**(A)** Schematics of molecular adsorption of MHMs in the active and inactive states. **(B)** SERS spectra were acquired from inactive and active MHMs after molecular enrichment (CV concentration: 1 × 10^−5^ M). **(C)** Raman intensity histograms of the five characteristic Raman peaks derived from **(B)**. **(D)** SERS spectra were acquired from active MHMs in different active states after molecular enrichment (CV concentration: 1 × 10^−5^ M). **(E)** Raman intensity histograms of the five characteristic Raman peaks derived from **(D)**.

Traditional SERS probes lacked propulsion and could only rely on passive diffusion to collect analyte molecules, so the detection time was limited by the diffusion rate of analyte molecules in the solution. MHM probes could accelerate the diffusion between silver nanoparticles and analyte molecules through active motion, improve the collection efficiency of analyte molecules, and thus reduce the collection time of analyte molecules and enhance SERS intensity. Here, the detection performances of the MHM probes on CV analytes under active and inactive states were compared. As illustrated in Figure 6A, the active state refers to the MHMs being in motion under an RMF, and the inactive state refers to the MHMs being at rest. CV molecules were collected by active and inactive MHM probes at the concentration of 1 × 10^−5^ M CV for 30 min. The Raman spectra of the two groups of probes are shown in Figure 6B. The red curve and the black curve represent the active and inactive MHM probes, respectively. The results show that the Raman intensity of the active MHM probes is approximately 170% that of the inactive probes (Figure 6C), which indicates that the active MHM probes can collect more CV molecules within the same time duration and has higher collection efficiency. Furthermore, Raman measurements were performed in different active states with the frequencies of 2, 5, and 10 Hz. The CV concentration for these tests was 1 × 10^−5^ M. As shown in Figures 6D,E, as the rotation speed of the MHM probes increased, the SERS intensity increased accordingly, indicating that the CV collection efficiency of the MHM probes in the active state was positively correlated with the rotational frequency of the MHM probes.

## 4 Conclusion

In this work, MHMs with high iron oxide content were fabricated and used as SERS probes. The HMs were prepared by UVL to achieve high consistency, high efficiency, and low cost. Iron oxide nanoparticles were grown in the HMs through *in situ* coprecipitation. The magnetic properties of MHMs were significantly enhanced by increasing the iron oxide content through continuous *in situ* coprecipitation cycles; after the first, third, and fifth cycles, the magnetization intensity of the MHMs reached 0.7, 3.3, and 12.0 emu/g, respectively. In the motion control test, the MHM under an 10-mT RMF showed a step-out frequency of 11 Hz and swimming efficiency of 0.1, which indicates that the MHMs were able to achieve good swimming performance than most of the previously reported magnetically actuated hydrogel-based microswimmers. In addition, the MHMs were able to maintain a steady rotation after reaching maximum velocity, indicating that the MHM can potentially adapt to different hydrodynamic conditions. To make MHMs capable of SERS detection, a layer of silver nanoparticles was uniformly grown on the surface of the MHMs through the silver mirror reaction. The silver nanoparticles enhanced the SERS signal of the analytes and helped facilitate the detection process with high sensitivity. In addition, when the MHM probes were actuated using an RMF, the active motion facilitated contact between the silver nanoparticles and the analyte and improved the detection efficiency of the MHM probes.

For the future study, we plan to design and fabricate magnetically actuated hydrogel-based achiral planar microswimmers with multiple layers of hydrogel using UVL. By incorporating multiple types of materials, it will be possible to create multifunctional MHMs for multiplexed detection of analytes.

## Data Availability

The original contributions presented in the study are included in the article/Supplementary Material; further inquiries can be directed to the corresponding author.
