# Supplementary material for: Magnetically-actuated hydrogel-based achiral planar microswimmers for SERS detection: In situ coprecipitation for continuous loading of iron oxide nanoparticles
Source: Front Bioeng Biotechnol. 2023 Mar 7;11:1086106. doi: 10.3389/fbioe.2023.1086106 (PMC10028090; doi:10.3389/fbioe.2023.1086106)
Supplement: Supplementary file 2 [file DataSheet1.pdf]

Supplementary Material

1. Supplementary Table

Table S1. A summary and comparison of different magnetically-actuated hydrogel-based microswimmers. The parameters are body length of microswimmers (L), step-out frequency (f), strength of rotating magnetic field (B), maximum swimming velocity ($V_{s,\,max}$), swimming efficiency ($\widetilde{U}_{s,max} = V_{s,\,max} / Lf$), maximum resultant velocity ($V_{r,\,max}$), and resultant efficiency ($\widetilde{U}_{r,max} = V_{r,\,max} / Lf$)

Magnetic Material Incorporation	Publication year	Images of microswimmers	Parameters
Surface coating	2018[1]	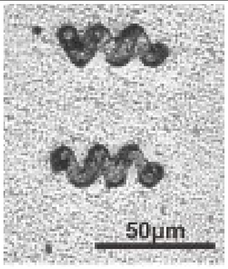	$B = 8$ mT<br>$L = 48$ μm<br>$f = 16$ Hz<br>$V_{s,\,max} = 16$ μm/s<br>$\widetilde{U}_{s,max} = 0.021$
Physical mixing	2019[2]	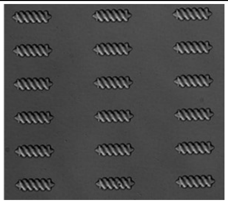	$B = 3$ mT<br>$L = 20$ μm<br>$f = 5$ Hz<br>$V_{s,\,max} = 3.36$ μm/s<br>$\widetilde{U}_{s,max} = 0.028$
Physical mixing	2019[3]	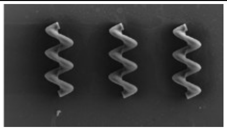	$B = 15$ mT<br>$L = 120$ μm<br>$f = 8$ Hz<br>$V_{r,\,max} = 82$ μm/s<br>$\widetilde{U}_{r,max} = 0.085$
Surface coating	2020[4]	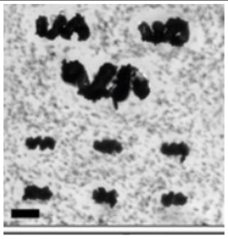	$B = 3$ mT<br>$L = 50$ μm<br>$f = 32$ Hz<br>$V_{s,\,max} = 80$ μm/s<br>$\widetilde{U}_{s,max} = 0.05$
Chemical conjugated	2021[5]	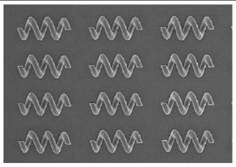	$B = 15$ mT<br>$L = 100$ μm<br>$f = 4$ Hz<br>$V_{r,\,max} = 85.7$ μm/s<br>$\widetilde{U}_{r,max} = 0.246$
Physical mixing	2021[6]	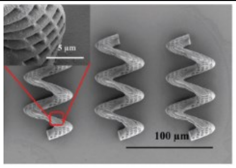	$B = 15$ mT<br>$L = 120$ μm<br>$f = 5$ Hz<br>$V_{r,\,max} = 69.1$ μm/s<br>$\widetilde{U}_{r,max} = 0.115$

In situ coprecipitation (This work)	2022	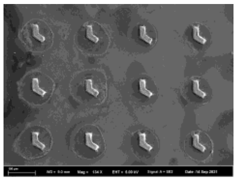	$B = 10$ mT<br>$L = 69$ μm<br>$f = 11$ Hz<br>$V_{s,\,max} = 74.8$ μm/s<br>$\widetilde{U}_{s,max} = 0.099$<br>$V_{r,\,max} = 214$ μm/s<br>$\widetilde{U}_{r,max} = 0.310$

Table S2. Assignments of vibrational bands in Raman and SERS spectra of crystal violet[7, 8]

Raman shift (cm^{-1})	Assignment
523	Bending mode of C–N–C
561	Out-of-plane aromatic C–C deformation
725	C–N–C symmetric stretching vibration
804	Phenyl-H out-of-plane antisymme
912	C–H out-of-plane bending modes
1172	C–H in-plane bending mode
1372	Stretching vibration of nitrogen and phenyl ring
1534	Phenyl-N antisymmetric stretching
1587	In-plane aromatic C–C
1618	In-plane aromatic C–C

2. Supplementary figures

Method: Cell viability and cell morphology

L929 cells were provided by the Stem Cell Bank at the Chinese Academy of Sciences. Cells were grown in MEM Complete Medium (contains 90% of no glucose MEM (GIBCO), 10% of (v/v) fetal bovine serum (FBS, GIBCO), and 1% of (v/v) penicillin/streptomycin (10,000 U/mL, GIBCO)). Cells were maintained at 37 °C in a humidified atmosphere of 5% CO_2 (v/v). Cell Counting Kit 8 (CCK-8) was obtained from Dojindo (Japan). AO/PI Double Stain Kit (BB-4142) was from BestBio Biotechnology (Shanghai, China). Other reagents were purchased from Sigma unless otherwise noted.

CCK-8 was used to quantitatively determine the cytotoxicity of samples. The hydrogel microswimmers were diluted using the cell culture solution, and the diluted concentrations were 1×10^7, 1×10^6, 1×10^5, and 1×10^4 pieces/L. The supernatants were obtained after 24 h of leaching in the refrigerator at 4 ℃ and sterilized by filtration. The cells were inoculated in 96-well plates (1×10^5 cells/well). After 24 h, 10% CCK-8 solution was added to the cell culture medium and inoculated at 37 °C for another 2 hours. The OD s at 450 nm was obtained through a multi-functional full wavelength microplate reader (Infinite 200 pro, Tecan Austria GmbH, Austria).

The live/dead staining assays were performed using BB-4142. Cells were seeded in 96-well plates at a density of 1×10^5 cells per well. Then different samples were added to corresponding wells. After 24 h of incubation, cells were washed twice with PBS. Afterward, 100 µL of the AO/PI Double Stain detection working solution was added following the manufacturer's instructions. After 15 min of incubation, cells were washed twice with PBS solution. AO can stain the nucleus of all cells (live cells and dead cells) through complete cell membranes, showing green fluorescence; PI can only pass through the incomplete cell membranes of dead cells, and stain the nucleus of all dead cells, showing red fluorescence. When the two dyes exist in the nucleus, under the appropriate ratio of AO and PI, the two dyes undergo resonance energy transfer, and the dead cells emit red fluorescence under the green channel. Finally, the living cells stained with AO ($\lambda = 525$ nm, green) and dead cells stained with PI ($\lambda = 630$ nm, red) were observed using an inverted fluorescence microscope (MJ6, Mingmei, China).

A

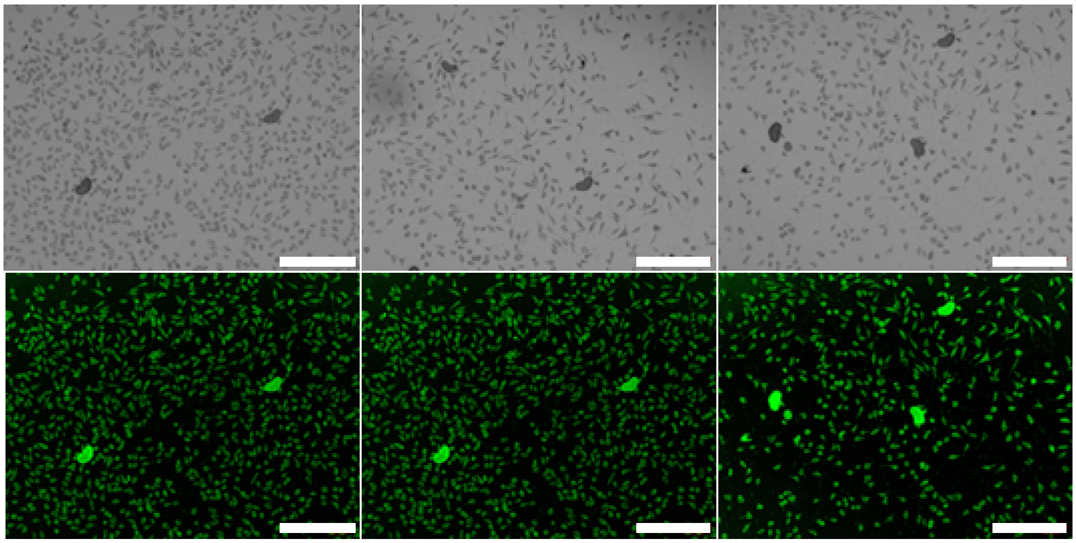

B

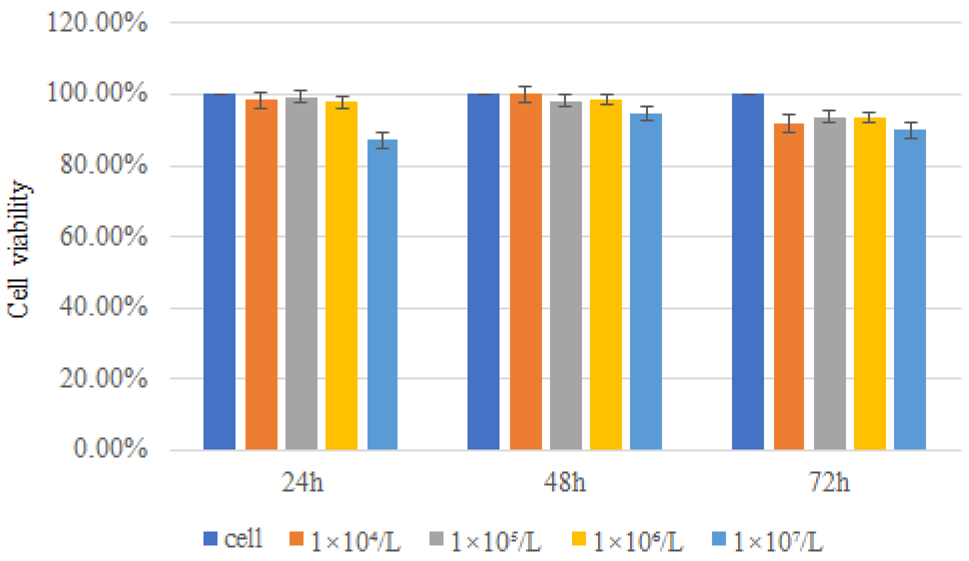

Figure S1. Cell viability experiments of the MHMs. (A) Optical and fluorescent images of live cell staining. The green color represents live cells. The scale bar is 200 µm. (B) Cell viability after 24 h, 48 h, and 72 h of incubation for different groups. The sample size was 5.

3. Supplementary movies

Movie S1 : A MHM swimming in deionized water.

Movie S2: Synchronous movements of two MHMs.

References

1. Wang, X.; Qin, X.-H.; Hu, C.; Terzopoulou, A.; Chen, X.-Z.; Huang, T.-Y.; Maniura-Weber, K.; Pané, S.;Nelson, B.J. 3D Printed Enzymatically Biodegradable Soft Helical Microswimmers. *Advanced Functional Materials* **2018,** *28* (45).
2. Ceylan, H.; Yasa, I.C.; Yasa, O.; Tabak, A.F.; Giltinan, J.;Sitti, M. 3D-Printed Biodegradable Microswimmer for Theranostic Cargo Delivery and Release. *Acs Nano* **2019,** *13* (3), 3353-3362.
3. Park, J.; Jin, C.; Lee, S.; Kim, J.Y.;Choi, H. Magnetically Actuated Degradable Microrobots for Actively Controlled Drug Release and Hyperthermia Therapy. *Advanced Healthcare Materials* **2019,** *8* (16).
4. Dong, M.; Wang, X.P.; Chen, X.Z.; Mushtaq, F.; Deng, S.Y.; Zhu, C.H.; Torlakcik, H.; Terzopoulou, A.; Qin, X.H.; Xiao, X.Z.; Puigmarti-Luis, J.; Choi, H.; Pego, A.P.; Shen, Q.D.; Nelson, B.J.;Pane, S. 3D-Printed Soft Magnetoelectric Microswimmers for Delivery and Differentiation of Neuron-Like Cells. *Advanced Functional Materials* **2020,** *30* (17).
5. Lee, H.; Kim, D.I.; Kwon, S.H.;Park, S. Magnetically Actuated Drug Delivery Helical Microrobot with Magnetic Nanoparticle Retrieval Ability. *Acs Applied Materials & Interfaces* **2021,** *13* (17), 19633-19647.
6. Park, J.; Kim, J.Y.; Pane, S.; Nelson, B.J.;Choi, H. Acoustically Mediated Controlled Drug Release and Targeted Therapy with Degradable 3D Porous Magnetic Microrobots. *Advanced Healthcare Materials* **2021,** *10* (2).
7. Liu, R.M.; Kang, Y.P.; Zi, X.F.; Feng, M.J.; Cheng, M.;Si, M.Z. The ultratrace detection of crystal violet using surface enhanced Raman scattering on colloidal Ag nanoparticles prepared by electrolysis. *Chinese Chemical Letters* **2009,** *20* (6), 711-715.
8. Lai, K.; Zhang, Y.; Du, R.; Zhai, F.; Rasco, B.A.;Huang, Y. Determination of chloramphenicol and crystal violet with surface enhanced Raman spectroscopy. *Sensing and Instrumentation for Food Quality and Safety* **2011,** *5* (1), 19-24.
